# Anemia in type 2 diabetic patients and correlation with kidney function in a tertiary care sub-Saharan African hospital: a cross-sectional study

**DOI:** 10.1186/s12882-016-0247-1

**Published:** 2016-03-19

**Authors:** Vitalis F. Feteh, Simeon-Pierre Choukem, Andre-Pascal Kengne, Daniel N. Nebongo, Marcelin Ngowe-Ngowe

**Affiliations:** Department of Internal medicine and Pediatrics, Faculty of Health Sciences, University of Buea, Buea, Cameroon; Health and Human Development (2HD) Research Group, P.O. Box 4856, Douala, Cameroon; Diabetes and Endocrine Unit, Department of Internal Medicine, Douala General Hospital, Douala, Cameroon; South African Medical Research Council, and University of Cape Town, Cape Town, South Africa; Department of Surgery, Obstetric and Gynecology, Faculty of Health Sciences, University of Buea, Buea, Cameroon

**Keywords:** Anemia, Chronic kidney disease, Diabetes, Estimated glomerular filtration rate, Hemoglobin, Cameroon

## Abstract

**Background:**

Anemia is common in diabetic patients and increases morbidity and mortality, but its burden has been less well characterized in sub-Saharan Africans. We determined the prevalence of anemia and investigated the related factors, with a particular focus on the role of declining renal function, in type 2 diabetic patients attending a tertiary health care institution in Cameroon.

**Methods:**

Hemoglobin (Hb) levels were measured in a consecutive sample of patients with type 2 diabetes, who reported for annual review at the outpatient section of the Douala General Hospital in 2013. Patients were classified as anemic according to the World Health Organisation criteria (Hb < 12 g/dl for females and Hb < 13 g/dl for males). Estimated glomerular filtration rate (eGFR) was calculated using the abbreviated Modification of Diet in Renal Disease Study Group formula. Determinants of Hb concentration and anemia were investigated using multivariable logistic regressions.

**Results:**

A total of 636 patients were examined including 263 (prevalence rate 41.4 %) who had anemia. The prevalence of anemia increased significantly with deteriorating kidney function, although up to 31.9 % of patients with normal kidney function had anemia. Compared with their non-anemic counterparts, anemic diabetic patients were older, had longer duration of diabetes, lower eGFR, higher prevalence of proteinuria and diabetic retinopathy (all *p* < 0.05). In multivariable logistic regressions, eGFR (*p* = 0.001) and presence of retinopathy (*p* = 0.023) were the independent determinants of prevalent anemia.

**Conclusions:**

The prevalence of anemia is high in type 2 diabetic patients attending referral institutions in Cameroon, including among those without chronic kidney disease. Routine screening for anemia in all diabetic patients may aid early identification and correction as appropriate.

## Background

Developing countries are experiencing an upsurge in the prevalence of diabetes [1], which is associated with a high risk of microvascular complications [2]. For instance, the prevalence of diabetes in Cameroon has risen from 1.1 % in 1997 [3] to 5.9 % in 2013 [1]. It is expected that the prevalence of long-term complications of diabetes will increase accordingly due to low awareness of the disease [1] and suboptimal management of those with diagnosed diabetes [4]. Among the chronic microvascular diabetes complications, diabetic nephropathy may present with anemia at different stages of deterioration of the kidney function [5]. Accordingly, anemia is a common feature in diabetes, with a reported prevalence of 13 % in a European population [6], 23 % in an Australian population [7] and up to 46.5 % in a Caribbean population [8].

Chronic kidney disease (CKD) does not explain all cases of anemia in diabetes, which seems to result from an interplay between several factors that include: declining kidney function, functional erythropoietin deficiency, nutritional deficiencies, chronic inflammation, iatrogenic causes, and infectious diseases [9]. As a consequence, anemia tends to occur early in diabetic patients, even in the absence of overt nephropathy, and it is more severe [10]. This is largely unrecognized by both physicians and patients despite the fact that anemia negatively affects the quality of life of diabetic patients [11]. Anemia is also a significant adverse prognostic factor for cardiovascular disease, and all-cause mortality in people with diabetes [12]. Early identification and correction of anemia has been shown to reduce the rate of progression and even delay the onset of some microvascular complications [13] as well as improve the quality of life of diabetic patients [14].

Current guidelines on the management of diabetes do not recommend routine screening for anemia. Although reports on the prevalence and predictors of anemia in diabetic patients exists elsewhere, such information is very rare in sub-Saharan Africa where other potential contributory factors like infectious and genetic diseases as well as nutritional deficiencies are very frequent and likely to worsen the burden of anemia. This study aimed to assess the prevalence and investigate the role of kidney function deterioration and other predictors of anemia in a population of type 2 diabetic patients in Cameroon.

## Methods

### Study design, setting and population

We conducted a cross-sectional study involving all consenting patients who were receiving chronic care for type 2 diabetes at the out-patient section of the endocrine unit of the Douala General Hospital (DGH), and covered a period of activity of 6 months from 1^st^ June to 31^st^ December 2013. DGH is a tertiary care hospital located in Douala, the economic Capital of Cameroon (approximately 2.8 million inhabitants) [15]. The endocrine unit of DGH is the main referral centre for endocrine diseases and diabetes in Douala. Patients with diabetes residing in Douala and surrounding regions were likely to receive care in this unit during the study period. Patients with diabetes receiving care in the unit undergo an annual evaluation that includes amongst others: a clinical evaluation, an assessment of diabetes control (HbA_1_c level), chronic complications (fundoscopy, retinography and retinal angiography where needed; dipstick proteinuria and serum creatinine), and cardiovascular risk factors assessment. The study received ethical approval from the Institutional review board of the Faculty of Health Sciences, University of Buea, and administrative clearance from the authorities of the DGH. Patients with diagnosed type 2 diabetes, irrespective of gender and aged 20 years or above, were included. All patients with a known hematologic disease, any acute condition, or those who received a blood transfusion in the preceding 4 months were excluded.

### Data collection and laboratory procedures

For each consenting patient, data were collected on medical and family history, and behavioral factors (smoking, physical exercise and alcohol consumption). The weight, height, blood pressure, waist and hip circumferences were measured using standardized methods. The body mass index (BMI) was calculated as weight (in kilograms)/height × height (in meters).

The hemoglobin level was obtained from the full blood count performed by an automated analyser (Ruby Cell Dyn^®^, Abbott). The Jaffe method was used to measure serum creatinine levels using a Roche – Hitachi Cobas C311^®^ analyser. Standard laboratory procedures were used in the measurement of HbA_1_c (immuno-turbidimetric method) and lipid profile (enzymatic method). Proteinuria was determined by dipstick.

### Definitions

Patients were classified as anemic according to the World Health Organization (WHO) criteria (Hb < 12 g/dl for females and <13 g/dl for males) [16]. A second definition of anemia was also used based on the suggested threshold of Hb < 11 g⁄dl (for both sexes) for the initiation of treatment with erythropoietin for anemia in chronic kidney disease [17]. Based on mean corpuscular volume (MCV), anemia was classified as microcytic (MCV < 80 fl), normocytic (MCV between 80 and 100 fl) or macrocytic (MCV > 100 fl). Mean corpuscular hemoglobin concentration (MCHC) was used to characterize anemia as hypochromic (MCHC <32 g/dl), or normochromic (MCHC ≥32 g/dl). Estimated glomerular filtration rate (eGFR) (ml/min/1.73 m^2^) was calculated using the abbreviated Modification of Diet in Renal Disease (MDRD) Study Group formula as eGFR =186 × [serum creatinine (mg/l)]^−1.154^ × [age (years)]^−0.203^ × 0.192 × (0.742 for female) [18]. Based on eGFR (ml/min/1.73 m^2^), patients were further classified into the five stages of chronic kidney disease (CKD) as follows: stage 1 (eGFR ≥90); stage 2 (eGFR between 60 and 89); stage 3 (eGFR between 30 and 59); stage 4 (eGFR between 15 and 29) and stage 5 (eGFR <15) [17, 18].

### Statistical analysis

Data were analyzed using SAS/STAT v.9.1 for Windows^®^ (SAS Institute Inc., Cary, NC, USA). We used the chi squared test for comparison of categorical variables, and the Student *t*-test or Kruskal-Wallis test for continuous variables. Results are presented as count (percentages), mean and standard deviation (SD) or median and 25^th^-75^th^percentiles as appropriate. Pearson correlation was used to assess univariate associations between continuous variables. Multivariable logistic regressions were used to identify determinants of anemia; odd ratio (OR) with 95 % confidence intervals (CI) are presented. A *p*-value <0.05 was used to characterize statistically significant results.

### Sample size estimation

The sample size was estimated assuming a prevalence of anemia of 40 % based on a previous study in people with diabetes in Nigeria [19], a precision of 5 % and a z value of 1.96. Based on these assumptions a minimum of 370 participants was required.

## Results

### General characteristics of participants

We included 636 type 2 diabetic patients (53.1 % males) aged 20 to 86 years (mean age 56.5 ± 10.6 years), with a median known duration of diabetes of 4 years (25^th^–75^th^ percentiles, 1–9). Compared with males, females were more likely to be overweight or obese (76.1 % vs. 70.1 %, *p* = 0.042), had poorer diabetes control based on mean HbA1c (8.5 % vs. 8.1 %, *p* = 0.046) and were less likely to be current smokers (4.4 % vs. 15.9 %, *p* < 0.001) (Table 1). Other baseline characteristics of the participants are summarized in Table 1.

**Table 1:** General characteristics of participants

Variables	Women (*n* = 298)	Men (*n* = 338)	*p*-value	Total (*n* = 636)
Mean age (years)	57.6 ± 11.5	55.6 ± 9.7	0.190	56.5 ± 10.6
Smoking	13 (4.4)	53 (15.9)	<0.001	66 (10.4)
Body mass index (kg/m^2^)	30.5 ± 6.6	28.2 ± 5.1	<0.001	29.3 ± 14.7
Overweight or obese (BMI ≥ 25 kg/m^2^)	228 (76.5)	237 (70.1)	0.042	465 (73.3)
Obese (BMI ≥ 30 kg/m^2^)	124 (44.6)	100 (32.1)	0.009	224 (38.4)
Waist circumference (cm)	97.3 ± 12.4	98.7 ± 12.9	0.167	98.0 ± 12.7
Hip circumference (cm)	108.4 ± 13.3	102.8 ± 10.7	<0.001	105.4 ± 12.3
Abdominal obesity^a^	252 (84.6)	198 (58.6)	<0.001	450 (70.8)
Systolic blood pressure (mmHg)	136.2 ± 21.1	137.7 ± 22.4	0.339	137.0 ± 21.8
Diastolic blood pressure (mmHg)	82.4 ± 12	82.1 ± 12.6	0.764	82.3 ± 12.3
Hypertension	188 (63.3)	206 (61.0)	0.542	394 (62.2)
Any BP-lowering medication	168 (56.4)	184 (54.4)	0.576	352 (55.3)
ACEI/ARB	145 (48.7)	163 (48.2)	0.575	308 (48.4)
Diuretics	104 (34.9)	114 (33.7)	0.654	218 (34.3)
Calcium channel blockers	72 (24.2)	68 (20.1)	0.129	140 (22.0)
Beta blockers	7 (2.3)	6 (1.8)	0.410	13 (2.0)
Use of metformin	244 (81.9)	268 (79.3)	0.696	512 (80.5)
Use of sulfonylureas	172 (57.7)	192 (56.8)	0.585	364 (57.2)
Use of Acarbose	6 (2.0)	8 (2.4)	0.335	14 (2.2)
Use of Insulin	31 (10.4)	37 (10.9)	0.383	68 (10.7)
HbA1c (%)	8.5 ± 2.6	8.1 ± 2.5	0.046	8.6 ± 2.5
Fasting capillary glucose (mg/dl)	150 ± 60	140 ± 60	0.646	150 ± 60
Any diabetic retinopathy	52 (22.4)	83 (30.4)	0.080	135 (26.7)
Any diabetic nephropathy	83 (31.6)	128 (40.9)	0.020	211 (36.6)
Any diabetic neuropathy	141 (51.1)	165 (52.4)	0.821	306 (51.7)
Serum Creatinine (mg/dl)	0.93 ± 0.36	1.22 ± 0.58	<0.001	1.08 ± 0.47
eGFR (ml/min/1.73 m^2^)	86.2 ± 30.4	86.2 ± 32.0	≥0.999	86.2 ± 31.7

Data are presented as number (%) or mean ± standard deviation;

*Abbreviations*: *ACEI* angiotensin-converting enzyme inhibitor, *ARB* angiotensin receptor blocker, *BMI* body mass index, *BP* blood pressure, *eGFR* estimated glomerular filtration rate, *HbA1c* glycated hemoglobin

^a^Abdominal obesity: waist circumference >80 cm in females or >94 cm in males

### Prevalence and distribution of anemia

The mean hemoglobin (Hb) levels were 13.2 ± 2.0 g/dl in males and 12.3 ± 1.5 g/dl in females (*p* < 0.001). According to the WHO criteria, 263 patients (41.4 %) had anemia. The prevalence was 42.6 % in females (*n* = 127) and 40.2 % in males (*n* = 136). Using the threshold recommended for intervention (Hb < 11 g/dl for both sexes), 91 patients (14.3 %), including 48 females (prevalence 16.1 %) and 43 males (prevalence 12.7 %), had anemia.

The average mean corpuscular volume (MCV) in the total population was 86.3 ± 7.7 fl. The difference in the distribution of mean corpuscular volume categories between patients with anemia and those without anemia was significant (*p* < 0.001), with anemic patients having more microcytosis and macrocytosis than non-anemic patients (Table 2). The average MCHC was 32.4 ± 2.7 in the total population. Low MCHC was more common in anemic than non-anemic participants, 103 (47.3 %) and 100 (38.3 %) respectively (*p* = 0.05) (Table 2).

**Table 2:** Comparison of red cell parameters categories between anemic and non-anemic patients

Variable	Anemia^a^ (*n* = 263)	No anemia (*n* = 373)
Normocytosis* (MCV 80–100 fl)	73.3	82.1
Microcytosis* (MCV < 80 fl)	21.4	15.1
Macrocytosis* (MCV > 100 fl)	5.4	2.9
Normochromia (MCHC 32–36 g/dl)	52.8	61.7
Hypochromia (MCHC < 32 g/dl)	47.3	38.3

^a^Anemia defined as hemoglobin concentration <12 g/dl for females and <13 g/dl for males

*p value < 0.05

### Kidney function and anemia

**Fig. 1:**
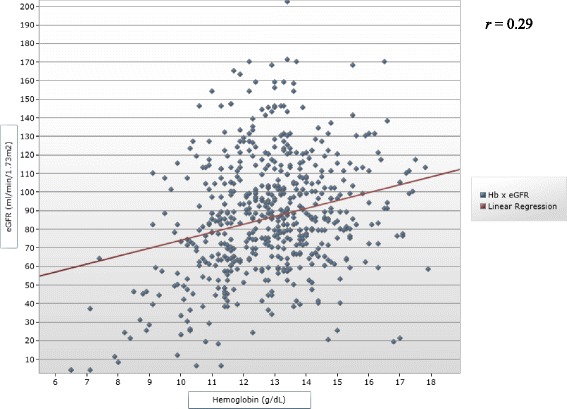
Correlation between the hemoglobin level and the estimated glomerular filtration rate. Squares represent individual hemoglobin levels of participants. The red line is the linear regression line

Proteinuria was positive in 68.4 % (*n* = 180) of patients with anemia as compared with 57.6 % (*n* = 215) of patients without anemia (*p* = 0.001). The mean eGFR of the 584 patients (91.2 %) with eGFR data was 86.2 ± 31.7 ml/min/1.73 m^2^. Patients with anemia had lower mean eGFR than patients without anemia (72.9 ± 35.7 vs. 89.6 ± 29.5 ml/min/1.73 m^2^, *p* < 0.001), and the Pearson correlation coefficient between the eGFR and the hemoglobin level was 0.29 (*p* < 0.001) (Fig. 1).

**Fig. 2:**
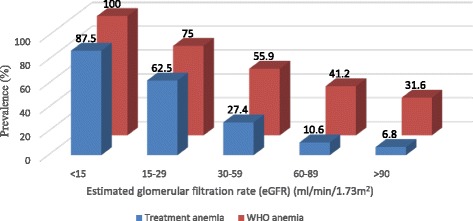
Prevalence of anemia by estimated glomerular filtration rate categories. Red bars represent anemia based on the World Health Organization definition; blue bars represent anemia based on threshold for intervention. Numbers above the bars denote the prevalence of anemia in each category of estimated glomerular filtration rate

Two hundred and fifty (42.8 %) participants had normal kidney function (eGFR ≥90 ml/min/1.73 m^2^) among whom 80 (31.9 %) were anemic compared with 160 (48.0 %) in patients with any CKD (*p* < 0.001). As kidney function deteriorated, the prevalence of anemia increased (Fig. 2). Compared with patients with normal eGFR, patients with CKD (eGFR < 90 ml/min/1.73 m^2^) of any degree were about two times more likely to have anemia at levels where treatment was required.

Among the 108 (18.5 %) patients with chronic renal failure (CRF) (eGFR < 60 ml/min/1.73 m^2^), 40 (37.0 %) had anemia at levels requiring therapeutic intervention, about four times higher than the 9.7 % in patients without CRF (*p* < 0.001).

### Other determinants of hemoglobin levels and anemia

Correlation coefficients between continuous variables and hemoglobin levels are shown in Table 3. Age, known duration of diabetes, systolic and diastolic blood pressure levels and serum creatinine were inversely correlated with hemoglobin levels, while waist and hip circumference, fasting glucose, HbA1c, and triglycerides were positively correlated with hemoglobin levels.

**Table 3:** Correlation between hemoglobin level and other variables

Variable	*r* (Pearson)	*p*
Age	−0.10	0.017
Duration of Diabetes	−0.18	<0.001
Systolic BP	−0.11	0.005
Diastolic BP	−0.10	0.011
Waist Circumference	0.15	<0.001
Hip Circumference	0.10	0.040
eGFR	0.29	<0.001
Fasting Blood Sugar	0.16	<0.001
HbA1c	0.12	0.009
Triglyceride	0.15	<0.001
Serum Creatinine	−0.07	<0.001

*Abbreviations*: *BP* blood pressure, *eGFR* estimated glomerular filtration rate

In multivariable logistic regressions, only eGFR [OR: 1.01 (95 % CI 1.00–1.02) per unit higher eGFR; *p* = 0.001] and the presence of diabetic retinopathy [OR: 2.32 (95 % CI 1.39–3.85); *p* = 0.023] were independent determinants of anemia.

## Discussion

In this study, we found that two in five patients with type 2 diabetes had anemia. This high prevalence was mainly driven by diabetes complications, as the presence of retinopathy and declining kidney function were the independent determinants of anemia. We also observed that one third of diabetic patients with normal kidney function had anemia.

Anemia is a common finding in patients with diabetes and has a negative impact on the patient’s sense of wellbeing; it also impairs the ability to work, reduces the quality of life [20] and worsens cardiovascular health [12]. The prevalence of anemia in our study (41.4 %) was relatively higher than that reported in the general adult population in Cameroon (34 %) [21]. The prevalence of anemia in diabetic patients reported elsewhere is comparable to, or lower than our finding, depending on the level of development of the country, the geographical altitude and the age of the study population: 46.5 % in Caribbean population [8], 17 % in Ethiopia [22] and 12 [6] to 23 % [7] in Caucasians. The higher altitude in Ethiopia and their younger study population may account for the lower prevalence, whereas the high prevalence of infectious causes and nutritional deficiencies, asymptomatic inherited hemoglobinopathies, and suboptimal glycemic control in our population could explain the higher prevalence in the present study compared with Caucasians [23]. The high prevalence of microcytosis in our study suggests the importance of non-renal causes of anemia in diabetes patients in this setting. This may have implications on the management options. However, further research is needed to fully investigate the spectrum of the etiology of anemia in African populations with diabetes.

In Cameroon, the prevalence CKD is about 13.2 % in the adult population, with the rate being higher amongst rural dwellers, and risk factors profile being similar to those in Caucasian populations [24]. In accordance with previous studies [6–8, 10, 22, 25] our findings illustrate the negative role of renal function decline in the development and progression of anemia in patients with diabetes. However, the absence of overt kidney disease was not protective against anemia, as 31.9 % of patients with normal eGFR had anemia; this proportion is high when compared with findings in other populations−13 % in China [25]. This observation emphasizes the role of other aforementioned contributors to anemia in diabetic patients in Cameroon.

Our finding that retinopathy was a significant determinant of anemia is in line with previous reports in India [26]. This is probably because diabetes retinopathy is usually associated with diabetes nephropathy. However, anemia may in turn play a pervasive role on the microvascular complications [27]. Although HbA1c was not an independent determinant of anemia, patients with normal hemoglobin levels had significantly higher level of HbA1c than anemic patients, as observed by other authors [25]. This raises caution in interpreting HbA1c levels in diabetic patients in Africa, as anemia has also been shown to spuriously lower HbA1c [28].

We acknowledge the following potential limitations of our study. Since it was conducted in a tertiary care hospital, results may overestimate the actual burden of anemia in diabetic patients in primary healthcare. Also this study was not designed to identify the causes of anemia in diabetic patients, indicating the need for further research in this domain. The reticulocyte count and the role of chronic inflammation on prevalent anemia was not assessed, and the presence of hemoglobinopathies was not investigated. However, we had a relatively large sample size of routinely monitored type 2 diabetic patients and we used robust statistical tests to identify the determinants.

## Conclusions

In conclusion, the prevalence of anemia in the type 2 diabetic population in Cameroon is high, including in patients with normal kidney function. However, declining kidney function appears as the main determinant. Our findings suggest that patients with diabetes should be routinely screened for anemia, and have other non-renal causes checked. Further, the findings raise issues regarding the added economic burden to diabetic patients attributable to anemia, and the cautious interpretation of the HbA1c level in sub-Saharan Africans.

## Abbreviations

BMI: body mass index
CI: confidence interval
CKD: chronic kidney disease
DGH: Douala General Hospital
eGFR: estimated glomerular filtration rate
Hb: hemoglobin
HbA1c: glycated hemoglobin
MCHC: mean corpuscular hemoglobin concentration
MCV: mean corpuscular volume
MDRD: modification of diet in renal disease
OR: odds ratio
WHO: World Health Organization
